# Visual Sensor Placement Optimization with 3D Animation for Cattle Health Monitoring in a Confined Operation

**DOI:** 10.3390/ani12091181

**Published:** 2022-05-05

**Authors:** Abdullah All Sourav, Joshua M. Peschel

**Affiliations:** Department of Agricultural and Biosystems Engineering, Iowa State University, Ames, IA 50010, USA; sourav@iastate.edu

**Keywords:** livestock monitoring, camera coverage optimization, sensor placement, genetic algorithm

## Abstract

**Simple Summary:**

This paper introduces a new method of finding the best locations to place video cameras inside large cattle barns to monitor the behavior and health of the animals. Current approaches to livestock video monitoring rely on mounting cameras in the most convenient places for installation, but those locations might either be impractical for actual barns and/or might not capture the best views. This work showed that there is short list of the best placement options for the cameras to choose from which will provide the best camera views.

**Abstract:**

Computer vision has been extensively used for livestock welfare monitoring in recent years, and data collection with a sensor or camera is the first part of the complete workflow. While current practice in computer vision-based animal welfare monitoring often analyzes data collected from a sensor or camera mounted on the roof or ceiling of a laboratory, such camera placement is not always viable in a commercial confined cattle feeding environment. This study therefore sought to determine the optimal camera placement locations in a confined steer feeding operation. Measurements of cattle pens were used to create a 3D farm model using Blender 3D computer graphic software. In the first part of this study, a method was developed to calculate the camera coverage in a 3D farm environment, and in the next stage, a genetic algorithm-based model was designed for finding optimal placements of a multi-camera and multi-pen setup. The algorithm’s objective was to maximize the multi-camera coverage while minimizing budget. Two different optimization methods involving multiple cameras and pen combinations were used. The results demonstrated the applicability of the genetic algorithm in achieving the maximum coverage and thereby enhancing the quality of the livestock visual-sensing data. The algorithm also provided the top 25 solutions for each camera and pen combination with a maximum coverage difference of less than 3.5% between them, offering numerous options for the farm manager.

## 1. Introduction

### 1.1. Use of Visual Sensors in Livestock Monitoring

There are two major hardware operations in cattle health monitoring with computer vision: data collection and processing units. Data are often collected through sensors or cameras, followed by processing on a personal computer. From a literature review, the cameras used in previous computer vision in livestock monitoring studies can be divided into the following categories: depth cameras, digital cameras, RGBD (red–green–blue color information with per-pixel depth information) cameras, and Closed-circuit Television (CCTV) or surveillance cameras. The use of digital and CCTV or surveillance cameras has been dominant in livestock health monitoring. These have been used for live weight estimation, lameness detection, individual cattle identification, behavior monitoring, and the tracking of pigs and cattle [1,2,3,4,5,6].

The camera or sensor installation location also varies based on the purpose of the research and the structure of the livestock housing. Cameras with a wide field of view or 360-degree lens are often installed on the ceiling [1,7,8,9,10,11]. Such installation facilitates capturing the whole cell and does not have occlusion issues while cattle or pigs stand behind one another. The camera installed in the ceiling is mostly practiced in the lab or research environment, as the average commercial feedlot does not have enough ceiling height for such an installation.

In most cases, the digital RGBD and depth camera is installed on the livestock housing ceiling to collect images and videos for further analysis. The captured data has shown promising results in estimating live weight [1,12], individual pig and cattle identification [8,11], aggressive behavior detection [13], mounting behaviors detection [9], standing behaviors detection [14], and so on. In contrast, digital and surveillance cameras have also been installed to collect side-view videos and images. Multiple studies successfully detected lameness, locomotion, and cattle and pig feeding behaviors using such setups [15,16].

### 1.2. Camera Placement Optimization

Regardless of the type, the camera is a valuable tool in computer vision systems to record and transmit spatiotemporal data in image and video format. The system also provides real-time information on livestock’s movement, posture, and behaviors [5,10,11,17]. Livestock behavior data collection with camera and quantification is an important tool for welfare monitoring and related research. Jackson et al. recorded used an optical camera to record piglet pen for a specific length of time [18]. On the other hand, Heiderscheit et al. recorded behavioral data of steer in video format from the Beef Nutrition Farm [19]. In both studies, time spent on drinking, eating, lying down, and displacement were calculated by visually observing the recorded video. Thus, accurate placement of the cameras for such research, as well as livestock monitoring, is crucial.

The purchase and maintenance cost of a surveillance camera system is often expensive [20]. In addition, changing the location of a surveillance system after installation is also inconvenient [21]. Thus, proper camera layout must be determined beforehand to calculate the number of cameras and their locations to be installed and minimize modification costs [22].

Achieving maximum camera coverage by minimizing the number of the cameras with a set of constraints is a complex optimization problem; thus, numerous studies have been conducted in this domain [23]. The camera optimization problems are similar to the Art Gallery Problem (AGP). The AGP is a well-studied computational geometric optimization problem finding the minimum number of guards with their restricted viewpoint required to cover all parts of the gallery interior. It is assumed that the guards/sensors have a 360-degree visual angle and unlimited viewpoint [24]. However, the camera visibility is limited due to its field of view angle and limited visual distance. The multi-camera coverage calculation problem treats each camera coverage differently, and all camera coverage is merged to maximize the total coverage. This multi-camera coverage optimization belongs to the class of non-deterministic polynomial-time hard (NP-hard) combinatorial optimization problem. Thus, computational complexity is expected to solve large instances and deal with multiple objectives [25,26].

The earlier studies in camera placement optimization solely focused on maximizing fixed camera coverage for building and indoor monitoring while considering the region of interest as a 2D plane [23,24,26,27]. However, in application, the camera covers a 3D space, and optimizing camera coverage in such an environment is computationally more complex than optimizing in a 2D plane [27]. The research paradigm has recently been shifted towards maximizing camera coverage in a 3D environment while minimizing the overall project cost and meeting certain constraints [28,29,30,31]. The process is computationally expensive but proved to be useful in different domains.

Kim et al. [28] performed a hybrid simulation of camera placement optimization to monitor construction job sites where the primary objectives were to maximize the coverage and minimize the cost. The objectives were also subjected to a certain budget, minimum coverage, and accessibility to power and data transmission constraints. The research work provided three solutions for three camera combinations for different price levels for the stakeholders. However, the work used Microsoft Excel to design the job site, which does not offer full-featured 3D modeling of the objects for precise camera coverage calculation. On the other hand, the job site was modeled using blocks of 1 m in size, which is relatively large and cannot yield very precious camera coverage calculation. Albahri and Hammad [29] proposed a coverage calculation method with the same primary objectives, but constraints were mostly regarding limiting the camera’s position to specific locations, pan, and tilt angle. In that simulation-based study, building information modeling (BIM) software played a crucial role in calculating the camera coverage by deriving geometrical constraints (e.g., ceiling, walls, and columns) and instrumental constraints (e.g., vibration caused by heating, ventilation, and air conditioning system). The study was overly dependent on building information and required two different programs, BIM and Unity 3D, to work in harmony for coverage calculation.

Other research has shown promising results in camera placement optimization for maximizing multi-camera coverage in indoor spaces (e.g., residential buildings, metro stations, and hospitals) and outdoor areas (e.g., construction sites, open urban areas, traffic intersections, and open sea) [22,24,28,29,30]. Despite such a wide variety of studies conducted, to the best of our knowledge, no camera placement study has been conducted to date on the welfare monitoring of livestock in a farm environment. This study focuses on achieving two objectives: (i) simulation-based camera coverage calculation for a confined environment of cattle and (ii) camera placement optimization simulation for achieving optimum camera coverage in a given budge.

This paper is organized as follows. Section 2 discusses the development of a 3D model and camera coverage workflow, followed by a genetic algorithm implementation to achieve maximum camera coverage at a given budget. Results of camera coverage workflow and the implementation of genetic algorithm with different camera and budget combinations are discussed in detail in Section 3. Section 4 presents this study’s research findings and limitations, followed by our conclusion in Section 5.

## 2. Materials and Methods

### 2.1. A Case Study for Camera Coverage Calculation

#### 2.1.1. 3D Environment Creation

**Figure 1:**
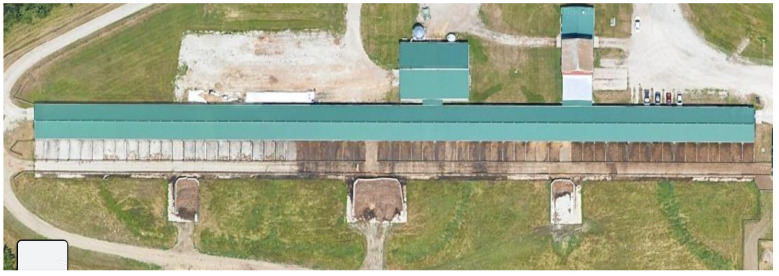
Aerial view of Beef Nutrition Farm at Iowa State University (GoogleMap).

**Figure 2:**
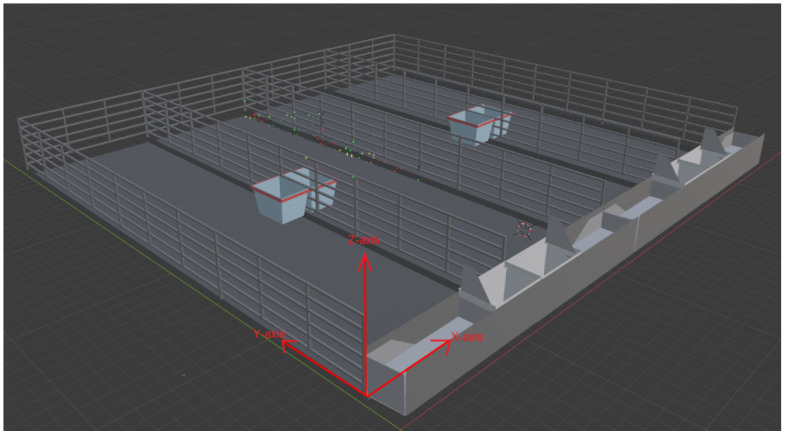
A sample confined farm scenario with four pens.

The first objective of this study was to calculate camera coverage for a given position and environment using camera properties. It has been observed that a majority of studies which have attempted to address camera placement optimization considered designing the environment in 2D space, whereas it is a 3D environment in reality. Calculating camera coverage using a 3D model close to an actual farm environment is also necessary for higher accuracy. In addition, some permanent physical obstructions are often overlooked in 2D and a poorly designed 3D scenarios. In our study, the 3D scene of the pen was created using the 3D animation software Blender. Blender is a free and open-source 3D creation cross-platform software that supports the entirety of the 3D pipeline—modeling, rigging, animation, simulation, rendering, compositing and motion tracking, even video editing and game creation [32]. It also supports Python scripting and access to Blender’s data, classes, and functions from its own Python modules (e.g., bpy and mathutils). This software was used in this study due to its scripting capabilities and simplicity for creating 3D scenes without expert knowledge. In the early stage of this study, measurements of a steer pen were collected from the Beef Nutrition Farm at Iowa State University (Figure 1). Six steers were usually housed in a 43 ft × 11 ft × 15 ft pen with a drinking trough and a feeding trough. The back of each pen was open to allow steer movement into the nearby open field, and on the front side there was a 9 ft open area for farmworkers and machine movement. A 3D farm scene with physical structures, fences, and dividing walls was created based on these measurements (Figure 2). The single pen was then copied and used to create the multi-pen scenario.

**Figure 3:**
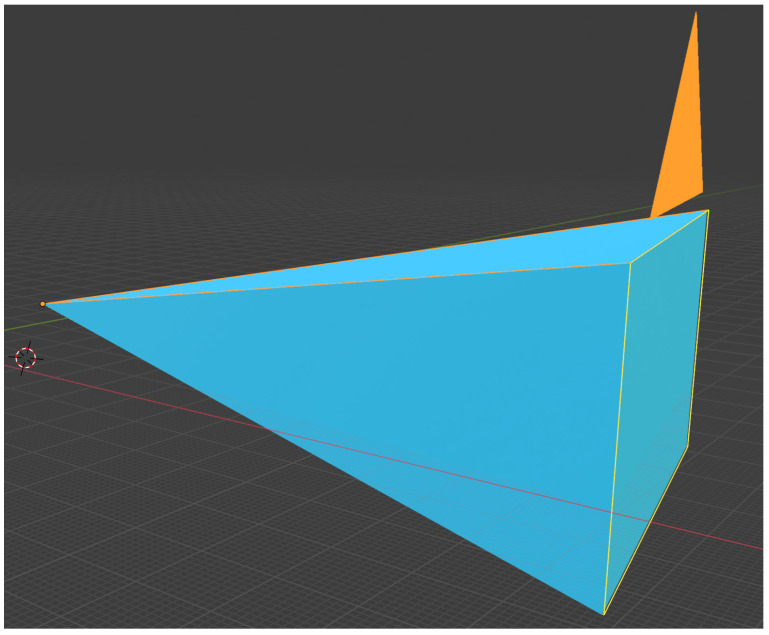
Duplicating the Blender camera properties on a cone shape Blender object with four vertices.

Blender has a default camera tool to adjust the location, view type, focal length, and field of view parameters. A 3D object with the exact shape and size of the camera was used to calculate the camera coverage. For the camera coverage calculation in this case study, we used the most common parameters of CCTV cameras available in the market. The cameras selected for this study had a field of view (FOV) of 76 degrees and 86 degrees. Once the camera was in place with the appropriate FOV, a camera shape object was created to represent the same physical properties of the Blender camera. A cone-shaped object with four vertices was created, and manually edited to have the exact shape and size of the Blender camera tool. This cone represented the total enclosed space recorded by each camera in a 3D environment. As shown in Figure 3, the yellow lines represent the Blender camera outlines, whereas the blue shape represents the cone created to represent the camera. The Blender objects are hollow, and if any object falls inside this hollow cone, it can be safely assumed that the object is visible on the Blender camera. The camera was enlarged in multiple folds, usually 60 ft in length, to check the visibility of all parts of a pen. As this study focuses on single and multi-camera setups, two cameras with 76 degrees and 86 degrees FOV were created, and their shapes were copied onto respective cones.

#### 2.1.2. Camera Placement

The camera placement locations were determined by the shape and size of the cattle pen. In our study, each pen had a size of 43 ft x 11 ft x 15 ft. The camera could be installed anywhere within the boundary of the pen. However, the back of each pen at the Beef Nutrition Farm was open for facilitating steer movement to the nearby field. Installing cameras at the back was not feasible, as the camera would be exposed to the rain and snow. In addition, cameras in such a position would not provide significant details of feeding behaviors, as feeding troughs were located at the front. Thus, we decided to use every other location except the backside as a viable camera placement location. The front side of the pen had a 9 ft clearance for instrument movement, and a camera could also be set up at 12 and 15 ft height on the opposite wall of the pathway. Viable camera location could be defined in 3D environment in terms of the *X*, *Y*, and *Z*-axis (Figure 2). Here, *X* and *Y*-axis represent the width and length of the pen, respectively. The *Z*-axis represents the height of the camera from the ground. Although there is an infinite number of *XY* locations throughout the boundary of a pen, a spacing between two viable camera locations was used to reduce the computational complexity. Initially, each camera location was set to be 3 feet apart on the *X*-axis and *Y*-axis. There are two possible *Z*-axis values: 15 ft and 12 ft. In addition, all the coverage calculations were completed starting at the (0,0) point and extended toward the *X* and *Y*-axis, as shown in Figure 2.

**Figure 4:**
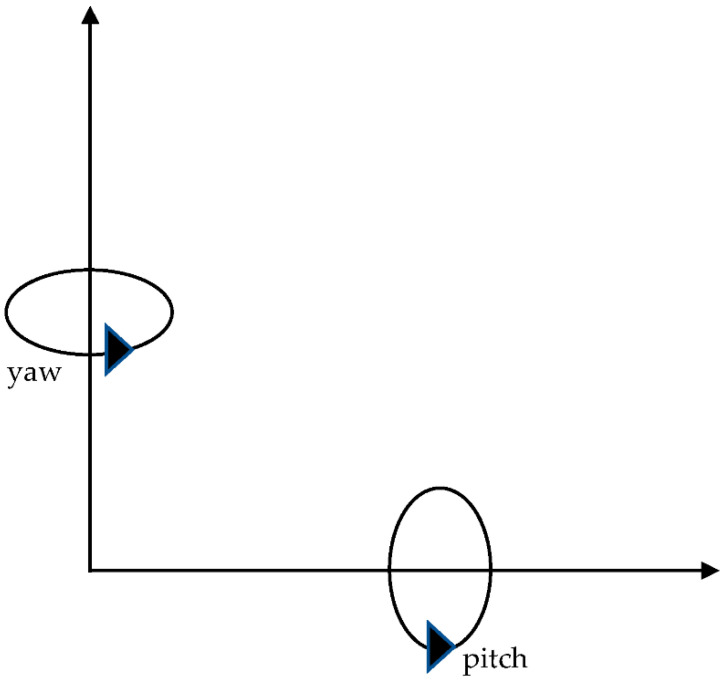
Yaw and pitch angle of the camera.

Each pose (*O*) represents camera coordinates (x,y,z), which indicates the camera’s exact location. The yaw and pitch angles represent the orientation of the camera (Figure 4). The yaw angle (γ) represents the camera rotation on the horizontal axis, ranging between 0 and 360 degrees. The pitch angle (ρ) represents camera rotation on the vertical axis, ranging between 0 and 360 degrees, as shown in Figure 4. The camera angles were limited to a specific range to only point it towards the region of interest or the cattle pen to avoid additional camera coverage calculation without significant coverage gain. The pitch angles were limited from 20 degrees and 60 degrees and the yaw angle was limited to the range 40 degrees to −40 degrees.

The area inside the cattle pen was the area of interest which could be assumed to be a large rectangular 3D object comprised of many smaller cubes. The center point of the cell (C_ijk_) expresses the location of the cell. In the 3D environment, i represents the *X*-axis value of the cell center, j represents the *Y*-axis value of the cell center, and k represents the *Z*-axis value of the cell center. If the center of the cell has a value of (4,9,5) then it is 4 units away from the (0,0) point on the positive x axis, 9 units away from the (0,0) point on the positive *Y-*axis, and 5 units above the floor. Each cell in the cattle pen was evaluated to check its visibility by the camera by checking the cell’s center point. In addition, calculating camera coverage for cells up to the roof of the pen is computationally expensive and will not provide any useful information. Thus, we used the average height of the steer to determine the height of the region of interest. In this case study, the height of the region of interest (ROI) was estimated to be 6 ft, whereas the approximate adult steer height was around 5 ft.

#### 2.1.3. Coverage Calculation

**Figure 5:**
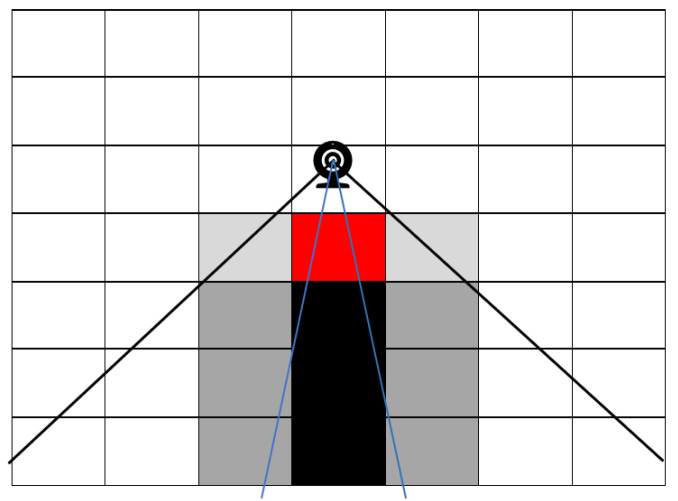
Occlusion due to physical structure; grey cells—partially visible, black cells—completely/mostly invisible, and red cell—physical obstruction.

Once the camera-shaped cone was positioned at a location (CM_ijkγρ_) with specific x, y, and z values and directed to a particular direction with the yaw and pitch angle, each cell’s center was examined to determine if it falls entirely inside the cone. The cone created using the camera’s dimensions was extended to a 60 ft length, so only cells visible to the camera were inside the cone. As physical structure of the pen such as the drinking trough, feeding trough, fence, pools, and other physical structures can block the view of the camera on the pen (Figure 5), ray cast, a native Blender function, was used to check for any visibility interruption of a particular cell. A ray was cast from the center of each cell (CC_XcYcZc_) toward the camera’s position (CM_XiYjZk_). The physical structures created using Blender were merged to form one mesh object. A cell was counted toward camera coverage if the ray did not intercept the physical structures of the feedlot. As shown in Figure 5, a cell could be partially visible to the camera but could not be counted toward camera coverage as the center of the cell is only considered for the total coverage calculation.

While camera coverage is expressed in the percentage of the cells visible to the camera, all the ROI cells might not have the same level of importance for specific research work. For example, in the Beef Nutrition Research Center of the Beef Nutrition Farm, the researchers are interested in the steer’s feeding and drinking behaviors for animal nutritional studies. Thus, setting the camera to monitor the steer approaching the feeding trough and eating is the primary objective. To facilitate this research, we counted the number of the weighted total cell on the total coverage. The complete working principle of this camera coverage calculation is provided below in Equation (1);

(1) $$C_{c}=\frac{\sum_{i=1}^{n}w_{i}\times c_{i\;}\;}{T_{c}}\times 100$$

Here, *C_c_* = percentage of the total weighted camera coverage on a specific location, *W_i_* = weight of a pixel in a particular group, *C_i_* = number of pixels on the camera coverage belongs to a specific group, and *T_c_* = number of total pixels inside the ROI.

The *C_c_* is expressed as a percentage, but the final number could be above 100% due to the different weight values assigned to the important pixels.

A cell visible to each camera was counted separately for multiple camera coverage calculations, followed by the union of two sets of camera coverage cells. A cell was counted toward the camera coverage calculation when it was visible to one or more than one camera, followed by a weighted coverage calculation.

### 2.2. Multi-camera Placement Optimization

Multiple camera placement was optimized using a genetic algorithm which is specifically designed to solve the multi-camera placement solution. In this method, a fixed number of cameras and their positions were evaluated and the highest camera coverage was delineated by considering a series of different camera locations. The camera position evaluation is based on multiple objectives set by the user, thus known as multi-objective genetic algorithms.

Genetic algorithms (GAs) were first proposed by Holland as a computational optimization model based on the principle of natural evaluation [33]. The two main ideas of evaluation those genetic algorithms borrowed are; (a) passing information from one generation to the next generation, also known as inheritance, and (b) competition for survival, or survival of the fittest. The main advantages of using genetic algorithms to solve optimization problems are adaptations and parallelism. Adaption works the best in finding a set of good solutions that might not be the best, and parallel calculation can be achieved without much communication overhead.

It is very challenging to achieve multi-objective goals in a real problem using the Genetic Algorithm, as objectives can conflict and lead to unacceptable results for a particular objective [32]. Konak et al. demonstrated two possible solutions based on previous studies in this domain to achieve acceptable objectives [32]. The first standard method is to move one objective into the set of constraints. The second method is to optimize a weighted sum of the objective functions. In this research, both approaches were followed. In the first case, the cost optimization objective function is moved to the set of constraints. In the second case, the total cost of the camera setup was multiplied by a weight followed by subtraction from the total coverage, which was being maximized. Thus, the higher cost setup was penalized more than lower cost setups for the same camera coverage. The goal is to select the genes with higher coverage but lower cost.

#### 2.2.1. Approach 1: Coverage Optimization with Budget Constraints

In the first case, the installation cost is used as the constraint rather than the objectives of the algorithm. The sole objective, in this case, is to maximize the coverage with a set of constraints

(2) $$\max\sum^{\;}C_{c1}\cup^{\;}C_{c2}\cup^{\;}C_{c3}\cup^{\;}\ldots \ldots \ldots \ldots \cup^{\;}C_{cn}+C_{awd}$$

*C_C_* represents the camera coverage by camera *C*. In each generation, some selected genes with the highest coverage was passed to the next analysis stage. $C_{awd}$ is the coverage awarded to the total coverage based on the secondary coverage and number of cells of the region of interest present on the coverage. The details of the secondary and awarded coverage are discussed in Section 2.2.4. The optimization function has the following constraints along with the common constraints discussed in Section 2.2.3.

(3) $$CIM_{1}+CIM_{2}+CIM_{3}\ldots \ldots \ldots +CIM_{n}\leq \;B_{c}$$

Here, *CIM* represents the installation and maintenance of the camera and *B_c_* represents the total budget of the farm manager.

#### 2.2.2. Approach 2: Weighted Sum of Coverage and Budget Optimization

Camera coverage maximization and cost minimization could be conflicting objectives. Thus, both objectives were unified into one with a fixed weight. Determining the most appropriate weight selection is challenging because the solutions can be changed.

(4) $$\max\sum^{\;}(C_{c1}\cup^{\;}C_{c2}\cup^{\;}C_{c3}\cup^{\;}\ldots \ldots \cup^{\;}C_{cn})*W_{T}+C_{awd}$$

Here, $W_{T}$ is the weight associated with the budget $CIM_{1}+CIM_{2}+CIM_{3}\ldots \ldots \ldots +CIM_{n}$ and$\;W_{T}$ is the weight for the associated cost, with a value between 0 and a specific percentage. If it represents the maximum possible cost, the penalty is a particular percent, 20% or 30% in this study. For zero-cost, the weight is 0; everything in between is interpolated based on the two extreme values.

#### 2.2.3. Common Constraints for Both Approaches

The abovementioned functions have the following constraints

(5) $$C_{c1}\cup^{\;}C_{c2}\cup^{\;}C_{c3}\cup^{\;}\ldots \ldots \ldots \ldots \cup^{\;}C_{cn}>CC_{min}$$

Here, *C_C_* represents the camera coverage by camera *C* and *CC_min_* represents the minimum required total weighted camera coverage for each camera position combination to be considered acceptable. This function is added to eliminate the combination with very low camera coverage.

Each camera must have a minimum coverage above a certain threshold defined by the user based on the number of pens and cameras the manager is planning to use.

(6) $$C_{cn\;}\leq threshold$$

The other constraints involved were

(7) 1≤i≤n

where *i* is the number of cameras; the user can define the maximum number of cameras.

(8) 0≤yaw≤360

(9) $$\frac{fov}{2}\leq pitch\leq 90-\frac{fov}{2}$$

The camera should focus on the region of interest. When the camera is positioned at 0 degrees, one side will focus on the ground. On the other hand, when the camera is positioned at 180 degrees, the upper side will focus parallel to the ground. An angle more than 90 degrees will point the camera upward. Thus, the pitch angle is fixed between a certain value to position the camera toward the region of interest.

Each camera placement combination cannot have more than one camera installed at any given location.

(10) $$x_{1}y_{1}z_{1}\;\neq x_{2}y_{2}z_{2}\neq \;\ldots \ldots \ldots \ldots \ldots \;\neq x_{n}y_{n}z_{n}$$

#### 2.2.4. Camera Coverage Award

As shown in Equation (4), the final adjusted weighted camera coverage is considered for genetic algorithm optimization. The adjustments were made based on the number of cameras placed, capturing data from high-priority surveillance areas, and the percentage of overlapping camera coverage areas. A similar approach was followed by Altahir et al. to optimize a multiview surveillance system [33]. Multiple sensors can capture the same location in visual sensor placement optimization studies. The common area or cells on the coverage is known as common coverage or secondary coverage.

As the coverage of each cell is in binary format, the total coverage is calculated using a binary OR operator, and the common coverage or secondary coverage can be attained by using an AND operator. The cell number under the common coverage was used to award a certain percentage of coverage. In this study, the awarded coverage is a linear interpolation between 0 and total number of cells on the *ROI* divided by the number of cameras. If all cells on the *ROI* fall onto the secondary coverage, the award was the total number of cells divided by the number of cameras used in the optimization.

(11) $$0\leq C_{s}\leq \frac{ROI_{cc}}{C_{n}}$$

As mentioned earlier, often the camera or sensor placement has some specific objectives, for example monitoring the feeding behaviors. In such a case, the closer the camera to the food container, the better the visual data. The camera coverage is subject to a certain award if the regions of interest can be visualized closely. For this study, if the camera covers the region of interest from the maximum allowable distance (pen length), then the number of cells covered will be counted only once. If it covers from the lowest possible distance, the cells inside the region of interest will be covered a specific number of times based on the user input. In this study, the maximum award was five times. Everything else in between is interpolated based on the two extreme numbers.

(12) $$C_{awd}=C_{roi}+C_{s}\;$$

$C_{roi}$ is the number award for a specific region of interest camera coverage. $C_{s}$ is the secondary camera coverage award.

#### 2.2.5. Genetic Algorithm Implementation

The objective function of this problem is to maximize coverage per expenditure. The genetic algorithm developed in this study has the following steps.

1. Generate random camera location: Each camera position has six parameters; camera, x, y, and z location, yaw angle, and pitch angle. Each random gene created had six parameters. For a single pen, single-camera scenario, a camera can be placed in an infinite number of locations. To reduce the resources and time required, each camera parameter was subject to some limitations. In the *X* and *Y* directions, feasible locations for camera placement were 3 ft apart. In addition, the *Z*-axis height could be either 12 ft or 15 ft based on the physical structure of the cattle pen. On the other hand, the yaw and pitch angle also had limitations, as shown in Equations (8) and (9).

Each of the parameters was selected randomly to create n number of genes. Each gene had the following formats:

*Camera name + X + Y + Z + Yaw + Pitch*

2. Check gene fitness: Survival of the fittest is the main motto of the genetic algorithm. Each of the N-genes generated randomly in the first step was checked to see if it had the minimum percentage of the required coverage. The user provides the threshold value, in this case, 100 cells. In a given location, no more than one camera can exist; multiple cameras at one location was also checked. Each camera parameter, or chromosome of the gene, was used to position a camera to a specific location and calculate the coverage and mean distance of the cells located inside the food container from the camera. Total adjusted camera coverage was calculated based on the total camera coverage and award and penalty coverage.

3. Offspring generation: Two parent genes were randomly selected from the pool of eligible genes to create offspring. Chromosomes/parameters were randomly selected from parents and merged.

4. Merging: A specific number of properties of the randomly selected genes were changed to prevent the algorithm from getting stuck at local minima or maxima. The default number of chromosomes to be changed in a gene was chosen as 4.

5. The highest scoring genes proceed to the next steps. The camera coverage for each gene was calculated. All genes were ranked based on the coverage optimization function. Only a specific number of genes with the highest adjusted camera coverage value were passed into the next steps.

6. Steps 2 to 5 were repeated for i iterations, which is defined by the user.

The above steps were implemented in Python 3.6. The program requires Blender’s native python libraries, mathutils, bpy, and bmesh, for camera placement in a specific location and coverage calculation.

**Figure 6:**
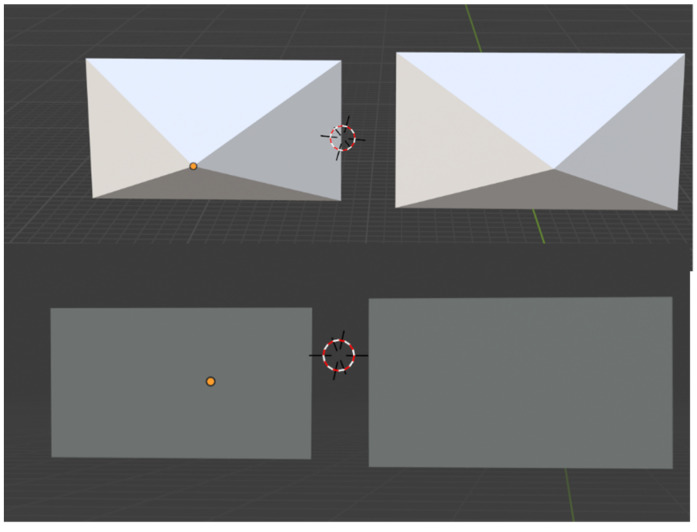
Camera view constructed in Blender Camera B (**left**) and Camera A (**right**) in back view (**top**) and front view (**bottom**).

The genetic algorithm was used to maximize the coverage while minimizing the cost for two different camera setups at the Beef Nutrition Farm at Iowa State University. The cameras were selected based on their price point and field of view. It was also assumed that camera A had FOV of 86 degrees and an installation cost of 200 USD. On the other hand, camera B had a 76 degrees FOV and an installation cost of 125 USD. The cameras were designed in Blender using the abovementioned properties on camera properties, followed by creating a cone-shaped camera. A comparison between the three cones shaped camera view are shown in Figure 6.

## 3. Results

### 3.1. A Case Study of Camera Coverage for Single Camera

A single pen at the Beef Nutrition Farm at Iowa State University equipped with a single camera was used as a case study for evaluation and validation of the working principle of this study. The camera properties used in this case study were determined by observing the most common properties found in the surveillance camera system available at Amazon.com, Inc. USA and priced below 500 USD. It is assumed that the camera had a resolution of 2560 × 1440 pixels and a field of view either 86 degrees (Camera A) or 76 degrees (Camera B). The following parameters were designed to create a camera object, as shown in Figure 6. The camera coverage calculations algorithm was executed, and the algorithm provided the top 20 results with the highest camera coverage. The best results for each cell size and each camera are provided in Table 1.

As shown in Table 1, the cell size of the region of interest was changed from 0.5 ft × 0.5 ft to 2 ft × 2 ft. The smaller cell size required significantly more time to calculate coverage. In addition, the camera with a larger FOV, camera A, provided significantly higher camera coverage than camera B with a smaller FOV. The result demonstrated a methodology for selecting the optimal location for a single camera with given parameters.

### 3.2. Multi-Camera Coverage Optimization with Genetic Algorithm

#### 3.2.1. Coverage Optimization with Budget as a Constraint

**Figure 7:**
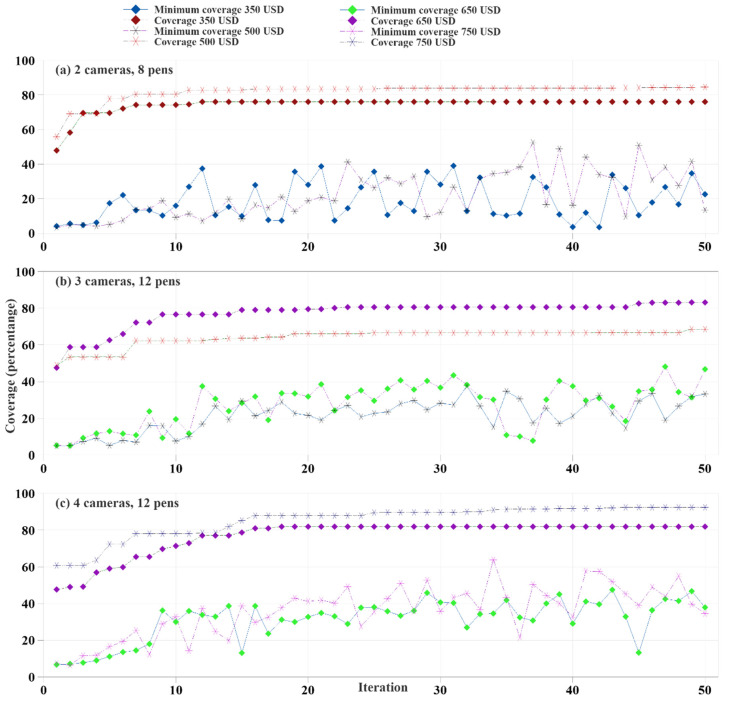
Camera coverage optimization for cameras at different budgets on eight pens setup. Subfigures (**a**–**c**) illustrate 2 cameras across 8 pens, 3 cameras across 12 pens, and 4 cameras across 12 pens, respectively.

Camera A and Camera B were used to find the best possible placement combination for optimal camera coverage. Using a genetic algorithm, two sets of camera and pen combinations were evaluated to find the best possible camera location. The first case used two cameras installed in the eight-pen setup as shown in Figure 7. In this setup, Camera A had a 86 degrees FOV and Camera B had a 76 degrees FOV, and both cameras had a resolution of 2560 × 1440 pixels. The genetic algorithm was used to find the optimal location for maximum camera coverage at different pen setups and two different budgets. In this scenario, a cell size of 1 was used.

The camera placement budgets were 350 USD and 500 USD, respectively. The optimal camera coverage was achieved after 34 and 45 iterations, respectively, as shown in Figure 7. Combined camera coverage was initially increased significantly, mostly for the first ten iterations. However, the improvement slowed down thereafter. There was a significant fluctuation in the lowest camera coverage among the different generations. The camera coverage and time required to run the algorithm were not always the same for each run, as genes were randomly created and edited in the different parts of the algorithm. The maximum camera coverage for 350 USD and 500 USD budgets were 76.1% and 84.3%, respectively. The camera coverage was increased for the 500 USD budget as the higher budget allowed use of camera A with a higher FOV.

The genetic algorithms were also used to maximize the coverage for four different budgets, 350 USD, 500 USD, 650 USD, and 750 USD, and two pen environments, eight pens and twelve pens. In the eight pens environment, the budgets and setups were 350 USD for two cameras and 500 USD for three cameras. In the twelve pens environment, the budgets and setups were 500 USD for three cameras, 650 USD for three cameras, 650 USD for four cameras, and 750 USD for four cameras. As shown in Figure 7, for all combinations, the camera coverage increased drastically over the first ten iterations, followed by a slight gain in camera coverage. For three cameras, the difference between maximum camera coverage of 500 USD for a two camera setup and 650 USD for a two camera setup was significant at 14.53%. This was due to the higher budgets allowed for selecting Camera A for all three allowed cameras. The four camera setups showed a drastic difference in maximum coverage with a higher budget. A budget increase of 100 USD allowed a gain of 10.5% of camera coverage. However, the four cameras with a 650 USD budget had 1.32% less maximum coverage than the three-camera coverage with the same budget, as the higher number of cameras forced the algorithm to select cameras with lower FOV. This difference shows that increasing the number of cameras does not always guarantee coverage gain after a certain point. The farm manager can efficiently decide on using a specific budget and number of cameras to achieve the desired coverage within their budget.

**Figure 8:**
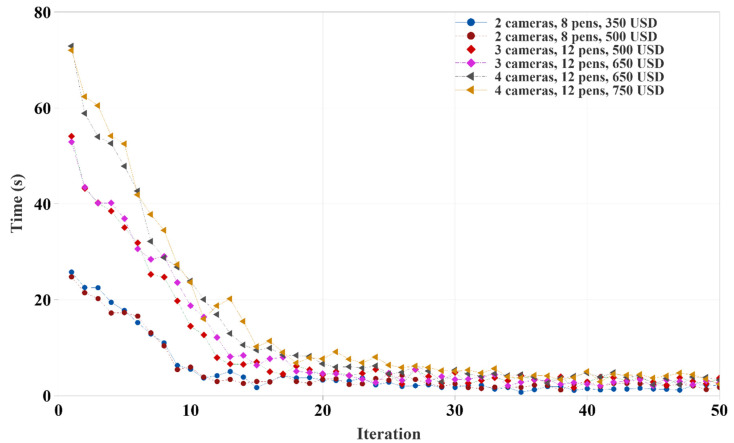
Time required to complete each iteration of different camera combinations in a given budget.

The time required to complete each iteration of camera coverage calculation was drastically reduced as the iteration progressed, as shown in Figure 8. The main reason for this was that our genetic algorithm saves the camera coverage for each camera combination in the Random Access Memory (RAM). The algorithm searches in the previous record of a given camera combination before calculating camera coverage. If it previously calculated the coverage, then recalculation of the same camera coverage was avoided to save time and computational resources. Thus, for two camera setups, the first five iterations took almost 20 s, the three and four cameras setups required 30 to 100 s, and then it came down from five seconds to a fraction of a second as the iteration progressed.

**Figure 9:**
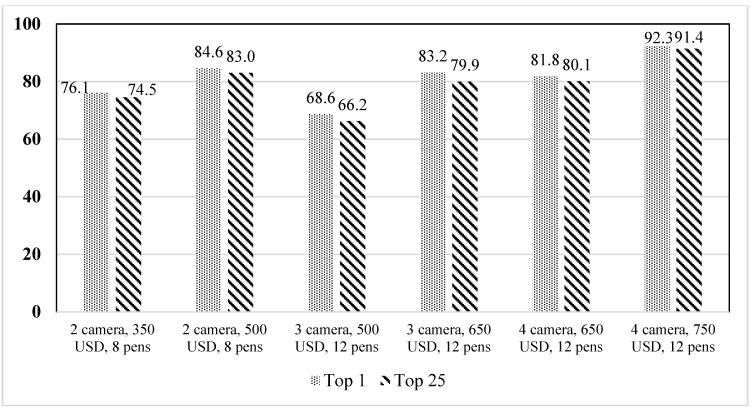
Difference in camera coverage between the top 25 camera placement solutions with budget as constraints.

Camera coverage solutions were ranked from high to low based on the percentage of area covered by the camera. The difference in the percentage of camera coverage between 1st and 25th on the ranked list is plotted in Figure 9. The figure illustrates that the difference between ranked 1st and 25th solutions was lowest at 0.7% for four cameras with a 650 USD budget for twelve pens and highest at 3.3% for three cameras with a 650 USD budget for twelve pens. Such a low difference shows that there were numerous camera placement options without significant percentage of coverage difference.

#### 3.2.2. Coverage Optimization with Budget Integrated into the Optimization Function

**Figure 10:**
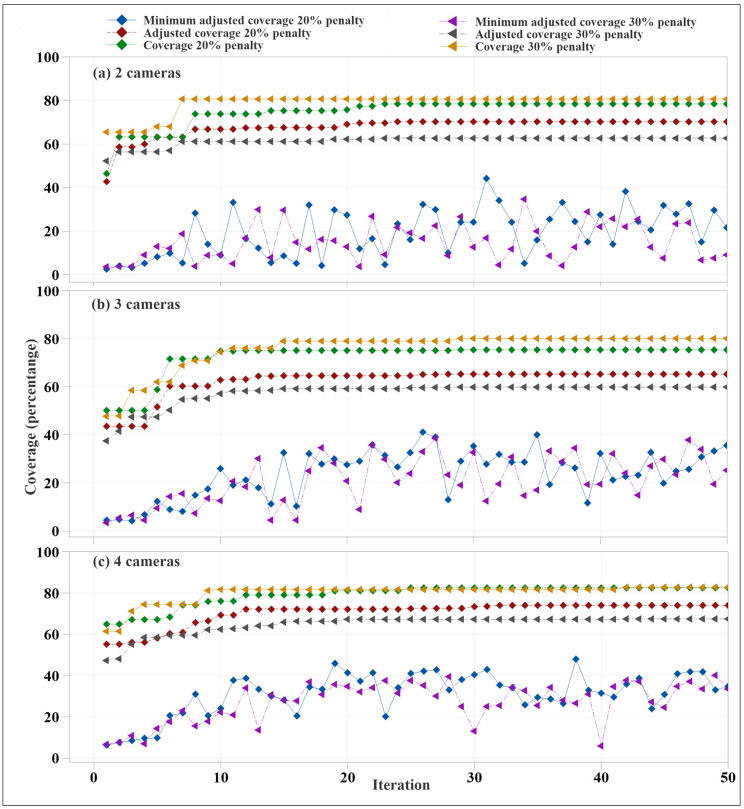
Camera coverage optimization for (**a**) eight pens–two camera coverage, (**b**) twelve pens–three cameras setup, and (**c**) twelve pens–four cameras setup.

The second approach to camera coverage optimization includes budget constraints in the optimization function. As described earlier in Section 2, the adjusted camera coverage was penalized based on the specific camera setup budget. The penalty was ranging from 0 to a specific percentage of the coverage. If the cost is zero, the penalty is zero; if cost is the maximum possible cost for a particular number of cameras, the penalty is a selected percentage. In this study, the maximum penalty amounts were selected as 20% or 30%, as shown in Figure 10. Users can define this percentage.

**Figure 11:**
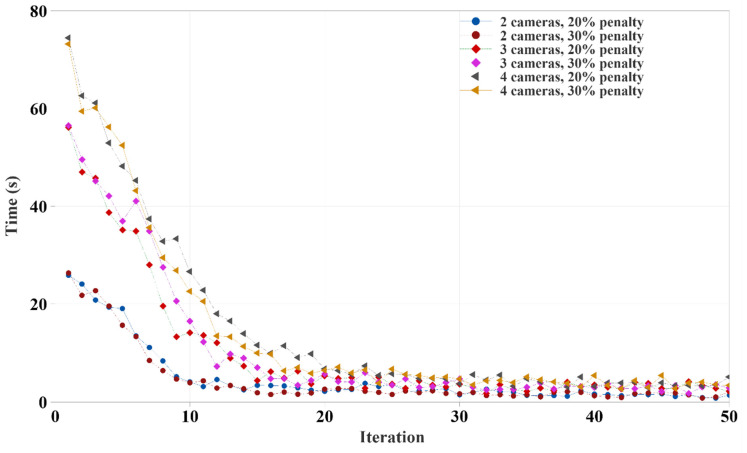
Time required to complete each iteration of different camera combination.

As shown in Figure 10, the eight pens–two cameras coverage, twelve pens–three cameras coverage, and twelve pens–four cameras coverage showed that the differences in total coverage in 20% cost penalty and 30% cost penalty were very low, less than 5%. This difference exists because the optimization algorithm was trying to maximize only the adjusted coverage. However, the adjusted coverage for the 20% penalty rate was significantly higher than for the 30% penalty rate coverage. It was also observed that the maximum adjusted and actual coverage did not reach their peaks simultaneously, because the algorithm focused on maximizing the adjusted coverage by changing the location and camera parameters. Like coverage optimization with a given budget, the coverage and adjusted coverage increased drastically at the beginning, mostly during the first ten iterations (Figure 11). The rate of additional coverage gains was very low afterward. Similar trends were observed for the three cameras–twelve pens and four cameras–twelve pens setups. The difference between maximum coverage was either small or zero for 20% penalty and 30% penalty. However, the difference between adjusted coverage was very significant in both cases.

In Figure 11, the time required to complete each iteration in the optimization follows a similar pattern to that shown in Figure 8. Size of the pen and the number of unique camera combinations or genes of the genetic algorithm to process dictated the required time. Initially, there was a relatively higher number of new genes or camera combinations. As optimization proceeded, the number of new genes or camera combinations not presented in previous iterations was low and were skipped to avoid duplicate processing. In addition, twelve pen combinations had 50% more cells than eight pen combinations, resulting in a higher processing time.

**Figure 12:**
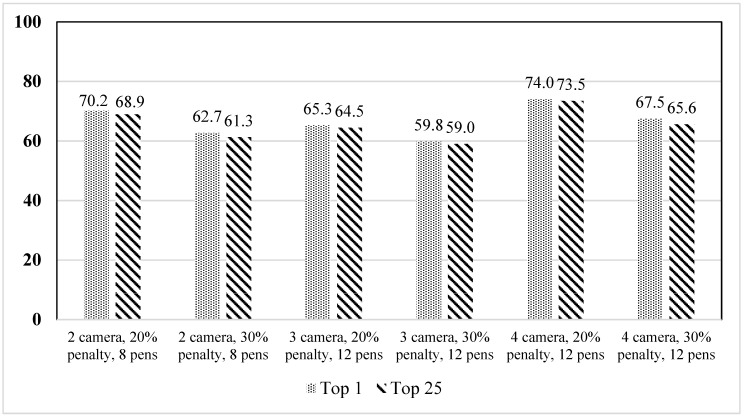
Difference in camera coverage between the top 25 camera placement solutions with budget integrated into the optimization function.

Figure 12 shows the difference between the 1st and 25th solutions raked from high to low based on the percentage of camera coverage provided by the genetic algorithm with budget integrated on the optimization algorithm. Figure 12 exhibited a trend very similar to that for the budget as constraint-based optimization illustrated in Figure 9. In this case, the lowest difference was only 0.5% for four cameras–twelve pens with 20% penalty, and the highest was 1.9% for four cameras–twelve pens with 30% penalty. Such a low difference in percentage can also offer numerous feasible solutions for camera placement.

## 4. Discussions

### 4.1. Findings

This study’s novel camera placement optimization methodology showed the efficacy of 3D animation software combined with an optimization algorithm to find the optimal solution with specific constraints in a large space. The results demonstrated that the optimal placement location could be derived for both a single camera and muti-camera setup in real-farm environment that takes the occlusion due to physical structure into account. The study pursued two different avenues of multi-objective genetic algorithms; coverage optimization with a given budget as a constraint and integrated budget function within the coverage optimization function. The results showed that the coverage difference in the 25 possible solutions, sorted based on percentage of camera coverage, was minimal, offering the user various options to choose from without significantly sacrificing total camera coverage. The study also addressed two major shortcomings of earlier studies in camera coverage optimization; taking the real 3D scenario into account and considering occlusion due to physical structures.

### 4.2. Limitations and Recommendations

The time required to complete this algorithm is its main limitation. The genetic algorithm was run with a cell size of 1 square ft, which is small compared to 1 square meter of some of the earlier studies. However, while a smaller cell size would yield higher precious coverage calculation, the time required for each iteration calculation increases significantly when the cell size is decreased. Occlusion was calculated based on the center of each cell, not the overall cell itself, which poses the risk of omitting a complete cell from the coverage for fractional occlusion due to the location of the cell center. While this study focused on optimizing camera placement in a relatively simple environment—a pen extending only on one side—this study can be adapted for a more complex environments with multistorey buildings and pens extending in different directions.

## 5. Conclusions

Surveillance data quality plays a pivotal role in cattle welfare monitoring with computer vision. In this study, a confined cattle farm environment was designed to determine the optimized camera location for the data collection. The multi-camera combination was solved by employing the genetic algorithm. Two approaches were followed to find optimal placement for maximizing camera placement; one with installation budget as a constraint and one with budget integrated into the optimization algorithm. The genetic algorithm showed that the optimal camera location could be determined within the first few iterations of camera coverage. In addition, the difference between the 1st and 25th result, when ranked from high to low based on percentage of camera coverage, also proved that the genetic algorithm could provide several optical camera locations to choose from. It was also observed that the FOV of the camera played the most crucial role in total coverage. The methodology also demonstrated that the approach can be adapted for camera coverage calculation in other domains with the versatile genetic algorithm and powerful Blender 3D software.

## Figures and Tables

**Table 1:** Calculated camera coverage of two different camera.

Pen	Camera	Cell Size	Camera Position	Optimal Camera Angles		Weighted Coverage (%)	Time Required
				Pitch	Yaw		
Single	A	0.5	7, −9, 15	60	0	99.69	613
		1	7, −9, 15	60	10	95.96	117
		2	3, −9, 15	60	−30	102.2	56.29
	B	0.5	11, −9, 15	60	30	97.05	1765
		1	0, −9, 15	60	−30	94.44	332
		2	11, −9, 15	60	30	99.05	128
Double	A	0.5	12, −9, 15	60	0	98.28	4236
		1	12, −9, 15	60	0	95.18	688
		2	0, −9, 15	60	−30	100	196
	B	0.5	12, −9, 15	60	10	91.43	1325
		1	12, −9, 15	60	10	90.82	218
		2	0, −9, 15	60	−30	97.4	190

## Data Availability

The data presented in this study are available on request from the corresponding author.
